# Reactogenicity and safety of the human rotavirus vaccine, *Rotarix*™ in The Philippines, Sri Lanka, and India: A post-marketing surveillance study

**DOI:** 10.4161/hv.29280

**Published:** 2014-08-01

**Authors:** Lulu Bravo, Amarjeet Chitraka, Aixue Liu, Jaydeep Choudhury, Kishore Kumar, Lennie Berezo, Leonard Cimafranca, Pallab Chatterjee, Pankaj Garg, Prasanna Siriwardene, Rommel Bernardo, Shailesh Mehta, Sundaram Balasubramanian, Naveen Karkada, Htay Htay Han

**Affiliations:** 1 National Institutes of Health-University of The Philippines Manila; Manila, The Philippines; 2 Max Superspeciality Hospital; Shalimar bagh; Delhi, India; 3 GlaxoSmithKline Vaccines; King of Prussia, PA USA; 4 Dr Jaydeep’s Clinic; Kolkata, India; 5 The Cradle; Bangalore, India; 6 West Visayas State University Medical Center; Jaro, Iloilo City, The Philippines; 7 Chong Hua Hospital; Cebu City, The Philippines; 8 Salt Lake Medical Center; Kolkata, India; 9 DNB Pediatrics; Consultant; Department of Neonatology; Sir Ganga Ram Hospital; New Delhi, India; 10 Family Clinic; Piliyandala; Colombo, Sri Lanka; 11 Davavo Doctors Hospital; Davao City, The Philippines; 12 GlaxoSmithKline Pharmaceuticals Pvt. Ltd.; India; 13 Kanchi Kamakoti Trust Hospital; Chennai, India

**Keywords:** safety, tolerance, vaccinology, India, infectious disease, reactogenicity, prescribing information, Sri Lanka, The Philippines, *Rotarix*™

## Abstract

Regulatory bodies in The Philippines, Sri Lanka, and India require post-marketing surveillance to provide additional safety data on *Rotarix*™ in real-life settings. In such studies conducted in The Philippines (November 2006 to July 2012; NCT00353366), Sri Lanka (November 2008 to August 2009; NCT00779779), and India (August 2009 to April 2010; NCT00938327), 2 doses of *Rotarix*™ were administered according to the local prescribing information (PI). The occurrence of at least Grade “2”/”3” solicited adverse event (AE) (fever, vomiting, or diarrhea), within 15 days in The Philippines or 8 days in Sri Lanka and India; unsolicited AEs within 31 days and serious adverse events (SAEs) throughout the study were recorded. Of the 1494, 522, and 332 infants enrolled in The Philippines, Sri Lanka, and India, 14.7% 14.9% and 12.7% infants, respectively recorded at least Grade “2”/”3” solicited AEs. The most commonly reported solicited AEs were irritability in The Philippines (32.2% post-Dose-1; 23.5% post-Dose-2) and India (23.0% post-Dose-1; 13.2% post-Dose-2), and fever (18.0% post-Dose-1; 20.2% post-Dose-2) in Sri Lanka. Unsolicited AEs were recorded in 24.5% (The Philippines), 4.8% (Sri Lanka), and 6.9% (India) of infants. Forty-one SAEs were recorded in the Philippines of which 6 (decreased oral intake with increased sleeping time and constipation; pneumonia, urinary tract infection, and intussusception) were considered by the investigators as causally related to vaccination. One vaccine-unrelated SAE occurred in a Sri Lankan infant. All SAEs resolved and the infants recovered. Two doses of *Rotarix*™, administered to healthy infants according to local PI, were well tolerated in The Philippines, Sri Lanka, and India.

## Introduction

In children below 5 y of age, rotavirus (RV) is the leading cause of severe gastroenteritis throughout the world and resulted in nearly 453 000 global deaths in 2008.[1] However, despite being a global disease, the vast majority of deaths (>85%) due to RV gastroenteritis occur in South Asia and Sub-Saharan Africa.[2]

According to estimates from the Asian Rotavirus Surveillance Network, nearly 45% of hospitalizations in Asia for acute diarrhea among children less than 5 y of age can be attributed to RV.[2] Among children in this age group, RV accounted for 31% of diarrhea-associated hospitalizations in The Philippines (2005–2006)[4] and 24% and 35.4% of diarrheal stool samples collected from hospitalized children in Sri Lanka (2005–2007) and India (2005–2006) were RV positive, respectively.[5,6]

The World Health Organization recommends the inclusion of RV vaccines in all national immunization programs,[7] particularly in countries where RV accounts for at least 10% of diarrhea-associated mortality in children below 5 y of age.[8]

Two currently available RV vaccines: *Rotarix*™ (GlaxoSmithKline Vaccines) and *RotaTeq*® (Merck and Co., Inc.) are well tolerated and efficacious against severe RV gastroenteritis.[9,10] Furthermore, in clinical trials, *Rotarix*™ was

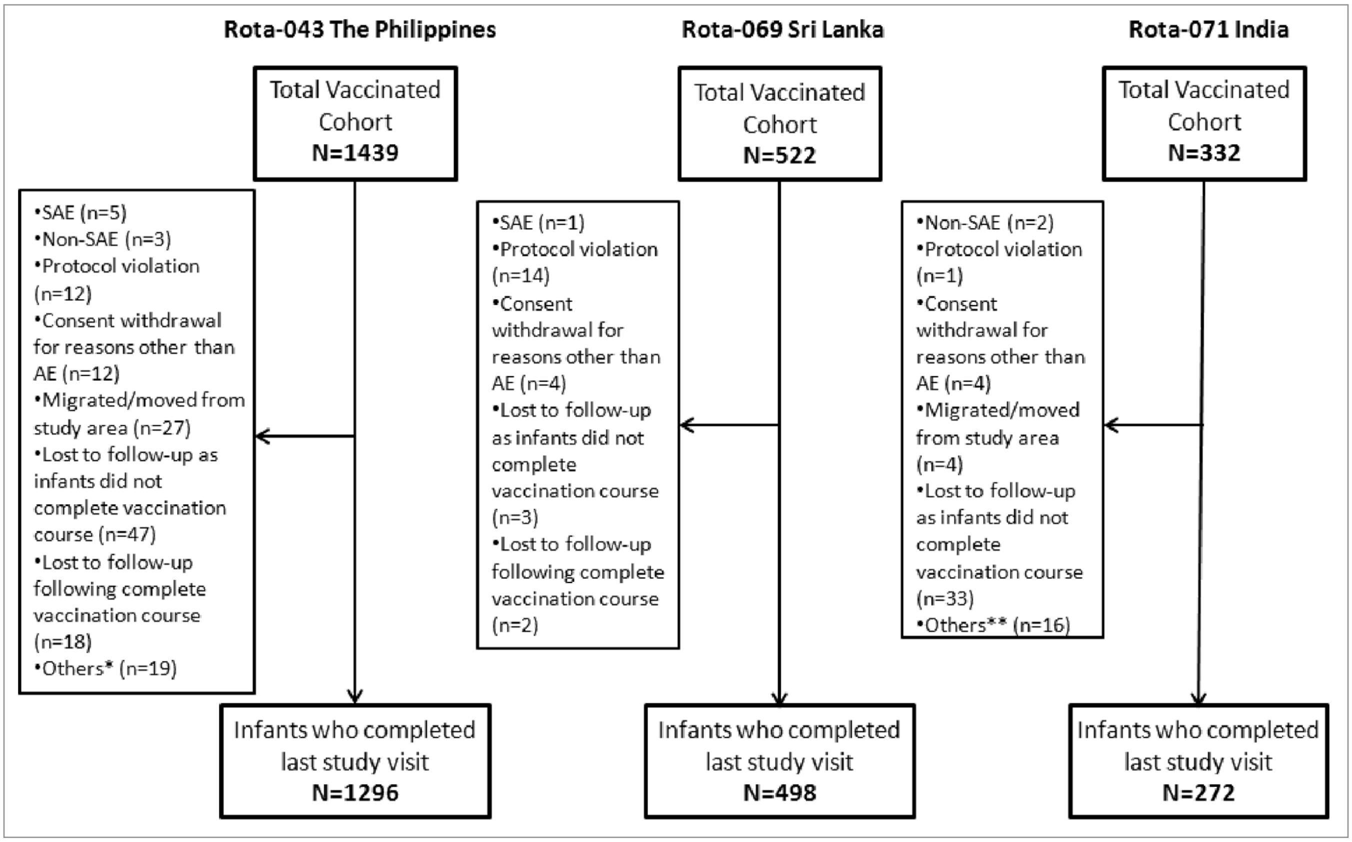

**Figure 1.** Study flow diagram. *Withdrawn due to: financial constraint; infant being over-age at the next follow-up visit; busy schedule of caregiver. **7 infants received Dose 2 of the vaccine at hospitals/clinics other than the PMS centers; 9 infants did not receive the vaccine as it was out of stock.

efficacious against severe RV gastroenteritis in infants up to 2 y of age in Europe (90.4%)[11] and Latin America (80.5%)[12] and up to 3 y of age in Asia (96.9%)[12] with no safety concerns. *Rotarix*™ was licensed in 2005 in The Philippines, 2006 in Sri Lanka and 2007 in India.

According to local regulatory requirements of: The Food and Drugs Directive, in The Philippines; The Cosmetics, Devices and Drugs Authority in Sri Lanka, and The Central Drugs Standard Control Organization, Directorate General of Health Services in India, post-marketing surveillance (PMS) studies are mandatory to provide additional safety data in real-life settings, once the vaccine is licensed for use. PMS studies were therefore conducted to evaluate the reactogenicity and safety of *Rotarix*™ when administered to healthy infants, according to the prescribing information (PI), in The Philippines, Sri Lanka, and India.

## Results

### Baseline characteristics

Of 1494, 522, and 332 infants enrolled in The Philippines, Sri Lanka, and India, 1296, 498, and 272 completed their respective local study (**Fig. 1**). In India, 67 infants had received first dose of *Rotarix*™ before entering the study.

The baseline characteristics of the enrolled infants are presented in **Table 1**. In The Philippines, the majority of infants were of East/South East Asian heritage. All Sri Lankan and more over half of Indian infants were of Central/South Asian heritage.

### Reactogenicity

*The Philippines*

Any AEs (solicited and unsolicited) was recorded in 57.4% (95% CI: 54.8–60.0) infants. At least one Grade "2" or "3" solicited adverse event (AEs: fever, vomiting, or diarrhea) was reported in 14.7% (95% confidence intervals [CI]: 12.9–16.7) of infants after either vaccine dose (**Table 2**). The incidence of any solicited AE was similar after Doses-1 and -2: irritability was the most frequently reported (32.2% [95% CI: 29.8–34.7] after Dose-1 and 23.5% [95% CI: 21.3–25.9] after Dose-2) (**Fig. 2**). At least one unsolicited AE was reported in 24.5% (95% CI: 22.3–26.8) of infants.

*Sri Lanka*

Any AE (solicited and unsolicited) was recorded in 48.7% (95% CI: 44.3–53.0) infants. At least one Grade "2" or "3" solicited adverse events was reported in 14.9% (95% CI: 12.0–18.3) of infants (**Table 2**). The incidence of any solicited AE was similar after Doses-1 and -2: fever was the most commonly reported solicited AE (18.0% [95% CI: 14.8–21.6] after Dose-1 and 20.2% [95% CI: 16.7–23.9] after Dose-2) **Figure 2**. At least one unsolicited AE was reported in 4.8% (95% CI: 3.1–7.0) of infants.

**Table 1.** Baseline characteristics (total vaccinated cohort)

| Characteristics | Parameters or categories | The Philippines n = 1439 | | Sri Lanka n = 522 | | India n = 332 | |
|---|---|---|---|---|---|---|---|
| | | Value/n | % | Value/n | % | Value/n | % |
| Age at Dose-1(ws) | Mean | 11.2 | - | 12.5 | - | 10.4 | - |
| | SD | 3.88 | - | 5.62 | - | 4.27 | - |
| Age at Dose-2 (wk) | Mean | 19.2 | - | 19.7 | - | 15.6* | - |
| | SD | 4.38 | - | 4.64 | - | 4.23 | - |
| Gender | Male | 747 | 51.9 | 255 | 48.9 | 177 | 53.3 |
| | Female | 692 | 48.1 | 267 | 51.1 | 155 | 46.7 |

*Age at Dose 2 not available for 60 infants as they withdrew from the study after Dose-1 in India. N, total vaccinated cohort; n, parameter/number of infants in the given category; %, (n/N) X 100; SD, Standard deviation.

*India*

Any AE (solicited and unsolicited) was recorded in 39.5% (95% CI: 34.2–44.9) infants. At least one Grade "2" or "3" solicited adverse event was reported in 12.7% (95% CI: 9.3–16.7) infants after either vaccine dose (**Table 2**). The incidence of any solicited AE was similar following Doses-1 and -2: irritability was the most frequently reported solicited AE (23.0% [95% CI: 18.1–28.6] after Dose-1 and 13.2% [95% CI: 9.4–17.8] after Dose-2) (**Fig. 2**). At least one unsolicited AE was reported in 6.9% (95% CI: 4.4–10.2) of infants.

**Serious adverse events**

*The Philippines*

Forty-one serious adverse events (SAEs) occurred in 33 infants. Six events in 4 infants (3 events of decreased oral intake by 50% with increased sleeping time and constipation in 1 infant; pneumonia, urinary tract infection, and intussusception [IS] in 1 infant each) were assessed to have a causal relationship to vaccination. All SAEs resolved and the infants recovered.

The case of life threatening IS occurred 1 d after the first dose of vaccine. The age of infant at the onset of IS was 15 wk. This infant received treatment with oral antibiotics and hydration therapy and was withdrawn from the study. The event was considered to have been: either possibly caused by *Rotarix*™ vaccination or due to acute gastroenteritis as a result of infectious diarrhea (not causally related to vaccination). Although this infant was lost to follow-up after withdrawal, the condition resolved at an unspecified date.

The other reported SAEs were considered as not causally related to vaccination and comprised: pneumonia, bronchopneumonia, bronchiolitis, asthma, hypersomnia, hypophagia, urinary tract infection, constipation, amoebiasis, amoebic dysentery, gastroenteritis, acute infectious diarrhea, roseola, viral rash, exanthema subitum. Apart from the case described below, all resolved and the infants recovered. The case report form from one infant who suffered 2 causally vaccine-unrelated SAEs (gastroenteritis and urinary tract infection, at 5 and 23 d post-Dose-1, respectively) was destroyed in a typhoon. Although these SAEs were recorded in the database, the infant was lost to follow up and the outcome of these SAEs is unknown.

*Sri Lanka*

Severe crying was reported in one infant 1 d after the first vaccine dose. This infant was hospitalized on the same day and underwent a surgery for undescended testes. This SAE was considered to be vaccine-unrelated and the infant recovered.

*India*

No SAEs were reported in India.

**Table 2.** , Occurrence of Grade "2" or Grade "3" adverse events (fever, vomiting, or diarrhea) during the solicited follow-up period (total vaccinated cohort)

| Doses | The Philippines | | | Sri Lanka | | | India | | |
|---|---|---|---|---|---|---|---|---|---|
| | N | n | % (95% CI [LL–UL]) | N | N | % (95% CI [LL–UL]) | N | n | % (95% CI [LL–UL]) |
| Dose-1 | 1439 | 147 | 10.2 (8.7–11.9) | 522 | 46 | 8.8 (6.5–11.6) | 265 | 29 | 10.9 (7.5–15.3) |
| Dose-2 | 1304 | 98 | 7.5 (6.1–9.1) | 501 | 50 | 10.0 (7.5–12.9) | 272 | 20 | 7.4 (4.5–11.1) |
| Overall/infant | 1439 | 212 | 14.7 (12.9–16.7) | 522 | 78 | 14.9 (12.0–18.3) | 332 | 42 | 12.7 (9.3–16.7) |

N, number of infants with at least one administered dose; n, number (percentage) of infants presenting at least one type of adverse event; %, percentage of infants reporting any AE; 95% CI, 95% confidence interval; LL: lower limit; UL: upper limit. Note: The solicited follow-up period was 15 d in The Philippines and 8 d in Sri Lanka and India.

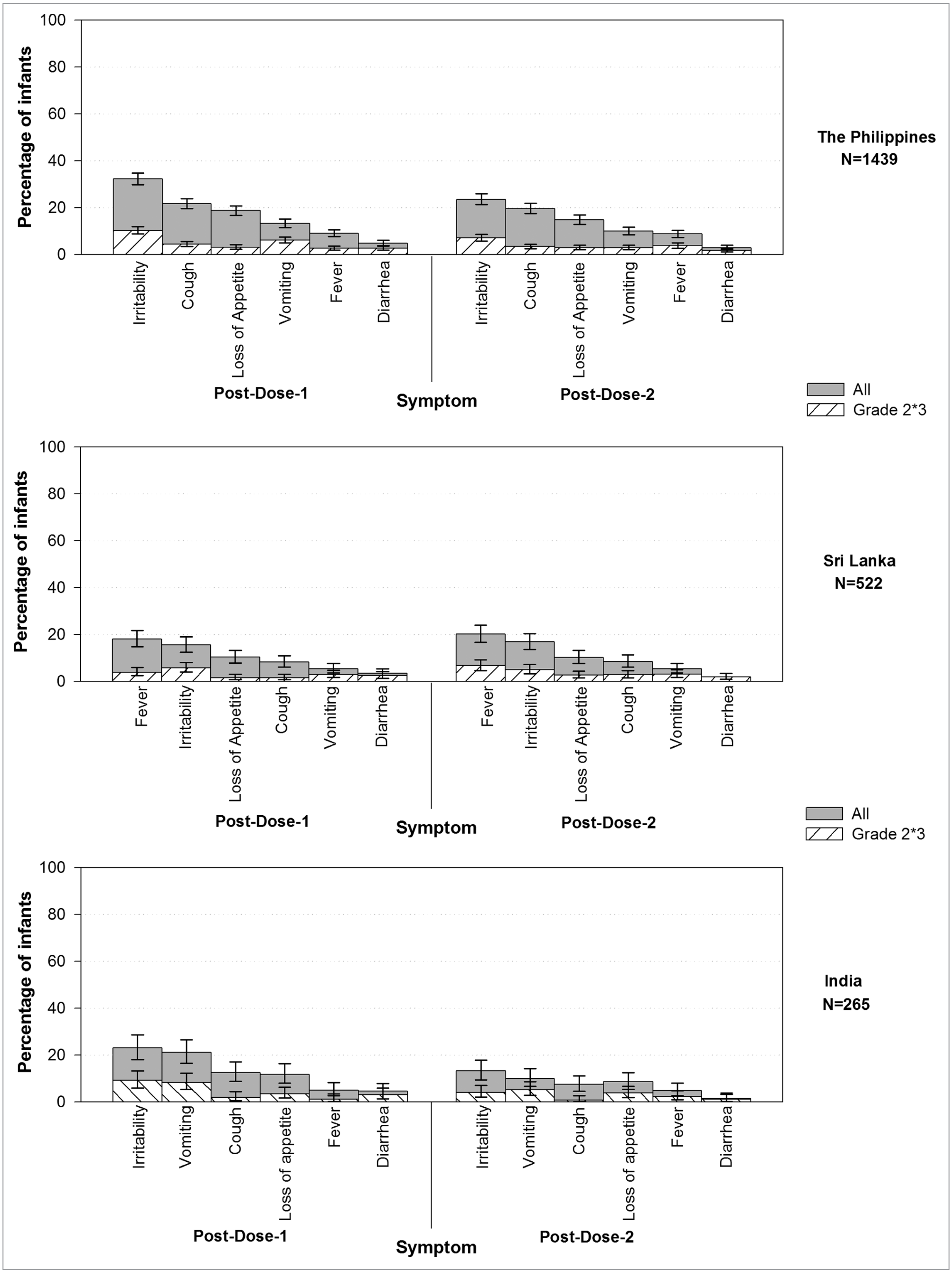

**Figure 2.** Occurrence of solicited adverse events after each dose of *Rotarix*™ (Total vaccinated cohort)

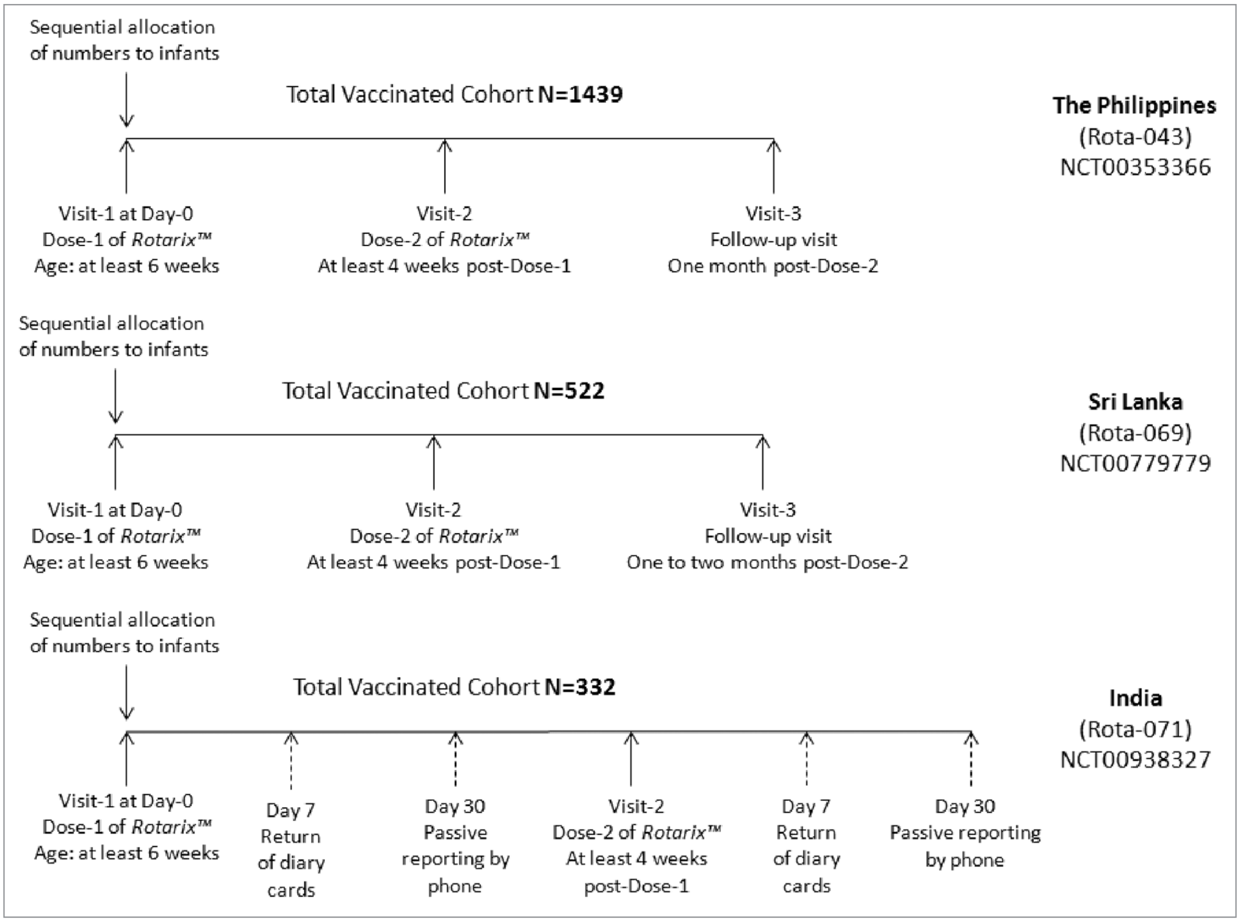

**Figure 3.** Study designs.

## Discussion

These 3 PMS studies evaluated the reactogenicity, tolerability, and safety of *Rotarix*™ when administered according to local PI to healthy infants in real-life settings. The results of these studies indicated that *Rotarix*™ had no safety concerns and was well-tolerated in healthy infants in all 3 countries.

The safety and tolerability of 2 doses of *Rotarix*™ has been previously documented from clinical trials conducted in various countries[10,14,15] and reported in an integrated safety summary of 8 double-blind, randomized, placebo-controlled, phase II and III studies.[16] This latter safety summary by Cheuvart et al.,[16] indicated that the reactogenicity and safety profile of *Rotarix*™ was similar to that of placebo, with no reported safety concerns.[16] These findings correspond to the results of the present PMS studies, where *Rotarix*™ was administered in a real-life setting.

One infant from The Philippines developed life-threatening IS 1 d after the first dose of *Rotarix*™, which the investigator considered to be possibly related to the vaccine. In a recent Mexican PMS study, a temporal increase in the risk of IS within 7 d of receiving the first dose of *Rotarix*™ was observed.[17] This translated to an estimated 3.7 (95.5% CI: 1.2–7.3) cases of IS per 100 000 person-years which could be attributed to *Rotarix*™ vaccination.[17] These observations were also consistent with another study conducted in Australia, where the relative risk of IS in infants during the first 7 d after the first dose of *Rotarix*™ was 3.41 (95% CI: 0.70–9.96).[18] Another case-control study in Mexican and Brazilian infants reported an increased risk of intussusception during the first 7 d after receiving the first dose in Mexico (odds ratio = 5.8 [95% CI: 2.6–13.0]) and during the first 7 d after receiving the second dose in Brazil (odds ratio = 1.9 [95% CI: 1.1–3.4]).[19] However in the present study, the infant with IS also suffered from infectious diarrhea which resulted in acute gastroenteritis and therefore the exact cause for IS could not be determined.

The difference in reporting rates of SAEs in the 3 countries could be due to the differences in attitudes of parents, culture, and referral patterns of parents, level of access to healthcare systems; the inclusion of symptoms such as decreased appetite and sleeping pattern as SAEs (parents sought hospitalization within the study period). However, the exact reasons remain unknown.

Virus shedding after vaccination with Rotarix™ was previously established, with peak shedding of vaccine virus around 7 d post-vaccination.[20,21] A review of published studies from various countries has also document similar findings, with up to 80% of infants shedding vaccine virus after the first dose and up to 29% after the second dose.[21] However, in the current PMS studies, collection of stool samples was not planned in the protocol and

**Table 3.** Definitions of Grade "2" or "3" solicited general adverse events

| Adverse event | Grade "2" intensity | Grade "3" intensity |
|---|---|---|
| Cough/runny nose | Interfered with daily activity | Prevented daily activity |
| Diarrhea | 4–5 looser than normal stools/day | ≥6 looser than normal stools/day |
| Irritability | Cried more than usual/ interfered with normal activity | Crying that could not be comforted/prevented normal activity |
| Loss of appetite | Ate lesser than usual/interfered with normal activity | Did not eat at all |
| Fever | Axillary temperature >38.0 to ≤39.0 °C | Axillary temperature >39.0 °C |
| Vomiting | 2 episodes of vomiting/day | ≥3 episodes of vomiting/day |

hence the virus shedding rates in the Philippines, Sri Lanka, and India remain unknown.

Our results need to be interpreted with caution due to the following limitations. First, since the sample size was low, it was not possible to identify all rare AEs. Second since these studies were PMS studies, the study designs were open, single group with no controls; the infants were not randomized. In addition, due to passive AEs and SAEs follow-up reporting after each dose of *Rotarix*™ and due to the difficulties in following-up infants visiting private clinics and outpatient wards, underreporting of AEs and SAEs is also acknowledged. Furthermore, the criteria for excluding infants from participating in the study were different in all 3 countries: in contrast to the infants in the Philippines, infants in India were excluded if they suffered gastroenteritis 7 d before vaccination and infants in both Sri Lanka and India were further excluded if they had a moderate or severe illness with or without fever. Finally, the only child who developed IS was lost to follow-up, because of which the duration of this SAE could not be determined.

## Conclusions

The routine administration of 2 doses of *Rotarix*™ according to local PI had no safety concerns and was well tolerated among healthy infants in The Philippines, Sri Lanka, and India. The observed reactogenicity and safety profiles were consistent with the *Rotarix*™ vaccine label.

## Methodology

**Study design and ethics**

Three open, single group, multi-center PMS studies were conducted in The Philippines (132 centers; November 2006 to July 2012; NCT00353366), Sri Lanka (5 centers; November 2008 to August 2009; NCT00779779), and India (11 centers; August 2009 to April 2010; NCT00938327). The study designs are illustrated in **Figure 3**. The 3 studies were each approved by the Independent Ethics Committees of their respective study centers and were conducted according to the principles of Good Clinical Practice and the Declaration of Helsinki. Parents/guardians of infants provided written informed consent before enrolment.

**Study population**

Male and female infants aged at least 6 wk at the time of first dose of *Rotarix*™, were included. Additionally in India, infants who had received a first dose of *Rotarix*™ before enrolment were also included as this study evaluated only the safety of the vaccine. These infants subsequently received the second dose of *Rotarix*™ during the study.

Infants were excluded from the studies if they had a history of allergy to any of the vaccine components; history of chronic gastrointestinal disease that included any congenital malformation of the gastrointestinal tract that was uncorrected; any contraindications as stated in the PI. Infants were also excluded if they: were participating concurrently in another clinical study; had suffered gastroenteritis in the 7 d preceding vaccination (India); or had a moderate or severe illness with or without fever (Sri Lanka and India).

**Vaccination**

Commercial lots of the vaccine were purchased locally by the infants' parents/guardians as per routine immunization practice in each country. Two doses of *Rotarix*™ were administered according to the PI of each country. The first dose was administered to infants at least 6 wk of age, followed by a second dose at least 4 wk thereafter. Both doses were completed by 24 wk of age.

All infants received routine vaccination with diphtheria, tetanus, acellular pertussis, hepatitis B, *Hemophilus influenzae* type b, and inactivated poliovirus vaccines concomitantly according to the Expanded Program on Immunization in their respective countries.

**Assessments**

Baseline demographic data was reported on the case report form by the investigators at the time of enrolment/first visit.

AEs (cough/runny nose, diarrhea, irritability, loss of appetite, fever, vomiting) were recorded on diary cards after each vaccine dose during the 15-d follow-up period in The Philippines and 8-d follow-up period in Sri Lanka and India. The intensity of solicited general Aes is described in **Table 3**.

Unsolicited Aes were recorded for a 31-d follow-up period after each dose and SAEs were documented throughout the study.

**Analyses**

The occurrence of at least one Grade "2" or Grade "3" AE (fever, vomiting, or diarrhea) during the solicited follow-up period in The Philippines (15 d), Sri Lanka, and India (8 d) after each vaccine dose was reported with the 95% CI (primary endpoint).

The occurrence of all solicited and unsolicited Aes within the specified follow-up periods following each dose were reported with 95% CI. All SAEs reported throughout the study period were described (secondary endpoints).

Statistical analyses for The Philippines study were conducted using Statistical Analysis Systems (SAS®) version 9.2 and 95% CI were calculated using StatXact-8.1. In Sri Lanka and India, the analyses were conducted using SAS® 9.1 and 95% CI were calculated using StatXact-Procs 7.0.

## Disclosure of Potential Conflicts of Interest

Authors S.B., P.C., L.R.C. and P.G. have no conflicts of interest to declare. N.K., S.M., H.H.H., and A.L. are employed by GlaxoSmithKline group of companies; H.H.H. and A.L. have stock options and H.H.H. received funding for travel/accommodations/meeting expenses. Author K.K. received grants for attending congress. L.B. is a member of GlaxoSmithKline Advisory Board for infectious disease prevention and an advisory in vaccination practices locally and regionally. L.B. received a minimal amount for patient's vaccine discount and research grants from GlaxoSmithKline group of companies, Pfizer and Sanofi; compensation for conducting lectures from GlaxoSmithKline group of companies and also received vaccine and other material donations for charitable activities. A.C. and J.C. received funding for CRA expenditure for data entry and eCRF and compensation for conducting lectures from the GlaxoSmithKline group of companies. P.S. obtained financial assistance for the secretarial work performed by the staff member of the institution from the GlaxoSmithKline group of companies. R.B. has attended conventions for which the travel was sponsored by GlaxoSmithKline group of companies. L.R.B. had no potential conflicts of interest to declare.

## Funding

This study was sponsored and funded by the GlaxoSmithKline Biologicals SA. GlaxoSmithKline Biologicals SA was involved in all stages of the study conduct and analysis. GlaxoSmithKline Biologicals SA also took in charge all costs associated with the development and the publishing of this manuscript.

## Acknowledgments

These PMS studies were sponsored by GlaxoSmithKline Biologicals SA, which also funded all costs associated with the development of the manuscript. The authors thank the infants and their families for participating and the investigators and study nurses from The Philippines, Sri Lanka, and India. The authors are grateful to the office secretaries and clinic assistants who followed up parents/guardians and reminded them to come back for their visits. The authors thank Harshith Bhat for medical writing; Geetha Subramanyam, Manjula K, and Lakshmi Hariharan for editorial and coordinating support in preparing this manuscript (all employees of GlaxoSmithKline group of companies) and Julia Donnelly (freelancer on behalf of GlaxoSmithKline Vaccines) for language editing.

## Trademark Statements

*Rotarix* is a trademark of GlaxoSmithKline group of companies.

*Rotateq* is a registered trademark of Merck and Co., Inc.
